# The Greek Version of Mini-Manual Ability Classification System (Mini-MACS): Translation and Reliability Study

**DOI:** 10.7759/cureus.30073

**Published:** 2022-10-08

**Authors:** Vasileios C Skoutelis, Niki Mastronikola, Argirios Dinopoulos, Eleni Skouteli, Zacharias Dimitriadis, Daphne Bakalidou

**Affiliations:** 1 Physiotherapy, Laboratory of Neuromuscular and Cardiovascular Study of Motion, Faculty of Health and Care Sciences, University of West Attica, Egaleo, GRC; 2 Physiotherapy, 'Attikon' University General Hospital, Chaidari, GRC; 3 Medicine, School of Health Sciences, National and Kapodistrian University of Athens, Athens, GRC; 4 Physiotherapy, ‘Mitera’ Children’s Hospital, Maroussi, GRC; 5 Third Department of Paediatrics, ‘Attikon’ University General Hospital, Chaidari, GRC; 6 Pediatric Neurology, ELEPAP - Rehabilitation for the Disabled, Athens, GRC; 7 Pediatric Neurology, ‘Mitera’ Children’s Hospital, Maroussi, GRC; 8 Physiotherapy, Health and Quality of Life Research Laboratory, Faculty of Health Sciences, University of Thessaly, Lamia, GRC

**Keywords:** translation, reliability, mini-manual ability classification system, correlation, cerebral palsy

## Abstract

Introduction: The Mini-Manual Ability Classification System (Mini-MACS) is an adaptation of the MACS for children with cerebral palsy (CP) aged 1-4 years, which classifies children’s performance to handle objects that are relevant to their age and development. The availability of a reliable Mini-MACS in Greek would allow for using it safely and properly in the clinical and research context of Greece. Therefore, the purpose of this study was to translate the original English version into Greek and examine its test-retest and interrater reliability.

Material and Methods: The English Mini-MACS was translated into Greek using the “forward-backward” method. Sixty-three children with CP, Gross Motor Function Classification System (GMFCS) levels I-V, aged 12 -50 months were included in the reliability study. Test-retest and interrater reliability were assessed using the interclass correlation coefficient (ICC). The association between Mini-MACS and GMFCS level ratings was also assessed using Spearman’s rho correlation coefficient (*ρ*).

Results: The translated version was easy to understand and use. The Greek Mini-MACS was found to have excellent test-retest reliability (ICC > 0.96) for both parents and therapists, good interrater reliability (ICC=0.89) between therapists and parents, and moderate-to-strong correlation with the GMFCS (*ρ *= 0.56-0.64, p < 0.0001).

Conclusion: The Greek Mini-MACS constitutes a user-friendly and reliable scale for use in the Greek population.

## Introduction

Cerebral palsy (CP) is the most common cause of childhood physical disability with 17 million people with CP worldwide, which leads to fine and gross motor impairments and activities limitations [1]. The current overall CP birth prevalence in high-income regions of Australia and Europe, including Greece, is 1.6 per 1000 live births [2], observing a drop by 25% compared to 2013 (2.1 per 1000) [3]. More than 50% of children with CP have manual ability problems [4]. Manual ability refers to the child’s ability to perform daily actions of handling objects using the hands, such as grasping, manipulating and releasing objects, including arm functions (e.g. supporting, hand positioning), whatever the strategies involved [5].

Manual Ability Classification System (MACS) is a simple five-level classification system used commonly in everyday clinical practice that classifies how children aged 4-18 years with CP use their hands while holding objects in daily activities [6]. Reliability and validity of the original Swedish and English versions of the MACS have been demonstrated [6], with the Greek MACS showing equally high interrater and test-retest reliability [7]. However, the low interrater reliability of the MACS to classify children aged 1-5 years with CP [8] led to the adaptation of the MACS for use in children with CP below 4 years of age, the so-called Mini-MACS [9]. The Mini-MACS differs from the MACS because of the need for the assistance of manual activities in children aged 1-4 years with CP and the nature of the objects they are expected to handle [9].

The availability of a reliable Mini-MACS in the Greek language will permit rehabilitation professionals in Greece to use it safely and properly both in their clinical practice and research. The primary purpose of the present study, therefore, was to translate the original English version of the Mini-MACS and to examine its test-retest and interrater reliability. Further, since there is no available information regarding the relationship between the Mini-MACS and Gross Motor Function Classification System (GMFCS), a secondary purpose was to examine their correlation with each other.

## Materials and methods

The present study consisted of two steps. The first step was a translation of the original English version of Mini-MACS leaflet in the Greek language. The second step consisted of an examination of test-retest and interrater reliability between therapists and parents of the final Greek version, and correlation with Greek GMFCS. This reliability study was approved by the Scientific and Ethical Council of the ‘Attikon’ University General Hospital, Chaidari, Attica, Greece (ΕΒΔ 499/21-09-2021). Written informed consent was obtained from all participating parents and therapists.

Development of the Greek version of Mini-MACS

Mini-MACS

The Mini-MACS is a further development of the MACS based on the same construct but adapted to make it appropriate for younger children aged 1-4 years. The children’s hand function is classified on a five-level scale from level I to level V, according to their ability to handle objects that are relevant for their age as well as their need for support and assistance in everyday manual activities and tasks. In the Mini-MACS, the general headings in levels I (handles objects easily and successfully), II (handles most objects but with somewhat reduced quality and/or speed of achievement), and V (does not handle objects and has severely limited ability to perform even simple actions) are identical to the MACS, but level III is just “handles object with difficulty,” and level IV (handles a limited selection of easily managed objects in simple actions) has “in simple actions” in place of “in adapted situations” [6, 9].

Translation into Greek

After receiving authorization from the original scale developer, Ann-Christin Eliasson, the English version of Mini-MACS was translated into Greek by four different translators through the use of “forward-backward” procedure, based on the guidelines for cross-cultural adaptation of therapy measures [10]. The forward translation into Greek was carried out by two Greek native independent translators with excellent knowledge of the English language. Both of them were physiotherapists but with no specific experience in the field of pediatric neurorehabilitation. The two forward translations were compared and synthesized into a common version by the manager of the translation process, who were academians and experts in pediatric neurorehabilitation. The resulting common forward translation was back-translated into English by a bilingual English-Greek translator, an expert in pediatric neurorehabilitation with no prior knowledge of the original version. Subsequently, the back translation was sent to the original developer of the Mini-MACS (Ann-Christin Eliasson) to evaluate whether the back-translated English version retained the meaning of the original English version.

After an agreement was achieved among Greek translation, Greek-to-English back translation, and the original English version, 10 experienced therapists in pediatric rehabilitation (five physiotherapists and five occupational therapists) and 10 parents of children with CP were invited to participate in cognitive debriefing interviews (pilot testing) by the main author and project manager in order to test the comprehensibility of the prefinal Greek Mini-MACS and identify any issues that cause confusion. Finally, the Mini-MACS Greek version was obtained.

Testing of reliability

To study the reliability of the Greek Mini-MACS an adequate [11] convenience sample of 63 young children, with all CP subtypes and all GMFCS levels, was included along with their parents. Inclusion criteria were young children, aged 1-4 years, with suspected or confirmed CP following pediatric neurological examination, based on neonatal cranial ultrasound (US) and MRI. Young children with identified genetic or metabolic conditions that mimic CP were excluded. The participants were recruited from public and private pediatric rehabilitation units across Athens metropolitan area (Attica).

All demographic information of the children, including gender, age, subtype of CP, was gathered from the health records at the recruiting center. The GMFCS levels were determined by each child's responsible experienced physiotherapist. The children were classified by one parent (mother or father) and one therapist (physical or occupational therapist) who was responsible for the child’s physical or occupational therapy at the rehabilitation center. The therapists knew or applied the Greek MACS [7] to the children with CP, aged 4-18 years. The majority of the participating therapists were physiotherapists (83.33%; n = 25), and just 16.66% (n = 5) were occupational therapists. In terms of parent participants 63.5% (n = 40) were mothers and 36.5% (n = 23) were fathers.

Procedure

After a brief verbal introduction and information about the Mini-MACS, the participants received the Greek version of the Mini-MACS leaflet and had time to read it and discuss any matter concerning the classification and rating process. Then therapists and parents independently rated their child’s ability to handle toys and objects according to the Mini-MACS levels. After about 7-10 days, the Mini-MACS was re-administered to all children by the same parent and therapist, in order to examine the test-retest reliability [12].

GMFCS

In the current study, the correlation of the Greek MACS with the Greek translation of the GMFCS was examined, which has substantial reliability (weighted kappa [κw] = 0.80, 95% confidence interval [CI] = 0.67-0.94) [13]. The GMFCS is a classification tool that allows an international age-based classification (under 2-18 years) of children with CP by severity level. The children’s gross motor function is classified on a five-level scale from level I (independent locomotion) to level V (dependent locomotion), according to their ability to do self-initiated motor functions such as sitting, changing postures, crawling, standing, and walking that are relevant for their age as well as their need for adult assistance and assistive mobility devices [14-15]. It has been found to be valid in many languages, including English (original version), Chinese, Korean, Persian, and Turkish [16].

Statistical analysis

The MacOS-based SPSS (IBM SPSS Statistics, Version 26.0, Chicago, IL, USA) software program was used in the data analysis of the study. Descriptive statistics (e.g., mean, standard deviation, median, frequencies, and percentages) were used for quantitative (age) and qualitative (gender, CP subtype, GMFCS level, Mini-MACS ratings) demographic characteristics of children. For analysis of interrater and test-retest reliability, an intraclass correlation coefficient (ICC) model 2.1 (two-way random effects model, absolute agreement) with 95% confidence intervals (95% CI) was used [17]. The ICC values of less than 0.50 can be referred to as indicating poor reliability, between 0.5 and 0.75 moderate reliability, between 0.75 and 0.90 good reliability, and greater than 0.90 excellent reliability [17]. Spearman’s rho correlation coefficient (ρ) was used to investigate the association between initial Mini-MACS and GMFCS level ratings. A Spearman’s correlation less than 0.20 was defined as very weak, 0.20-0.39 as weak, 0.40-0.59 as moderate, 0.60-0.79 as strong, and greater than 0.80 as very strong [18]. Statistical significance was set at p < 0.05.

## Results

Translation procedure

There were no semantic or conceptual difficulties during any part of the translation procedure. The developer of the original version confirmed that the back-translated version of the Mini-MACS was equivalent to the English with the only discrepancy being found in the double-meaning Greek word “παιχνίδι” (pehnidhi) that means both “toy” and “game.” The translator used inadvertently the word “games” in place of the word “toys” in the back-translated sentence “…children use their hands in order to handle objects, such as games…”.

The findings of the pilot test indicated that both therapists and parents had no difficulty understanding the instructions and descriptions of the Mini-MACS. All the participants agreed that the scale was easy to use and did not require any clarification. Therefore, the pre-final version was not in need of further adjustments and was deemed to be both comprehensible and cognitively equivalent to the original English version. As a consequence, the prefinal version was accepted as the final version of the Greek Mini-MACS. The Greek version of the Mini-MACS leaflet can be found free at https://www.macs.nu.

Descriptive analysis

For the reliability testing of the Greek version of the Mini-MACS, six cases were recruited from ‘Attikon’ University General Hospital, Chaidari, 17 cases from ‘ELEPAP-Rehabilitation for the Disabled’ charitable rehabilitation center, Athens, and 40 cases from private rehabilitation centers. The age range of the included young children was 12-50 months, with a mean age of 29.57 ± 12.81. Table 1 shows the results of descriptive characteristics of the children, including the rehabilitation units of recruitment.

**Table 1:** Demographics of the participating children with cerebral palsy at baseline. GMFCS, Gross Motor Function Classification System; NOS, (still) not otherwise specified; SD, standard deviation; UGH, University General Hospital

Characteristics	n=63	%
Gender		
Male	40	63.5
Female	23	36.5
Mean age, months (±SD, min-max)	29.57 (±12.81, 12–50)	
Cerebral palsy subtype		
Spastic quadriplegia	11	17.5
Spastic diplegia	14	22.2
Spastic hemiplegia	13	20.6
Dyskinesia	3	4.8
Ataxia	1	1.6
Unspecified (NOS)	21	33.3
GMFCS level		
I	24	38.1
II	21	33.3
III	7	11.1
IV	5	7.9
V	6	9.5
Unit of recruitment		
‘Attikon’ UGH	6	9.5
‘DEKA’ center	4	6,3
‘ELEPAP’ center	17	27
‘ENA’ center	10	15.9
‘Θeraπia’ center	11	17.5
‘Paidokinisi’ center	12	19.0
‘Specialized Therapy’ center	3	4.8

Reliability and correlation

The test-retest reliability was excellent for the parents (ICC2.1 = 0.97, 95% CI = 0.95 - 0.98) and for the therapists (ICC2.1 = 1.00).

The interrater reliability between therapists and parents was good with a reported ICC2.1 of 0.89 (95% CI = 0.82-0.93). There was disagreement for a total of 18 cases (29%), of whom 11 were rated a lower level (indicating higher ability) by parents, and five by therapists, while in the two remaining cases, parents classified their children at two lower levels. Of 18 disagreements between the classifications of parents and therapists, 9 (50%) occurred between levels I and II, 2 (11%) between levels II and III, 5 (28%) between levels IV and V, one (5.5%) between levels II and IV and one (5.5%) between III and V. The distribution of the Mini-MACS ratings by therapists and parents is shown in Table 2.

**Table 2:** Distribution of Mini-MACS by therapists and parents. Mini-MACS, Mini-Manual Ability Classification System

	Mini-MACS scores					
	I	II	III	IV	V	Total
Therapists	24	24	2	5	8	63
Parents	27	22	3	7	4	63

**Figure 1:**
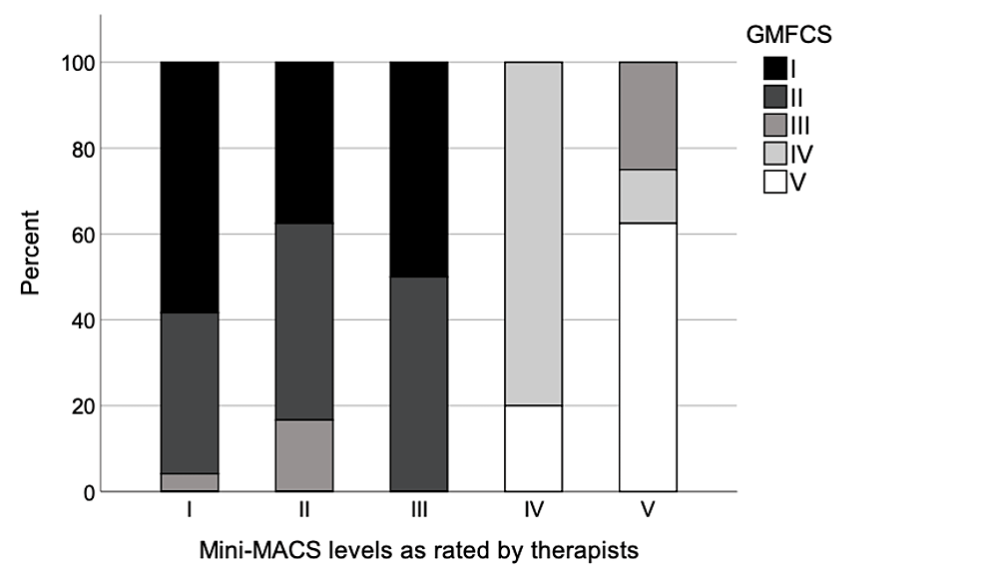
Percent distribution of the participated children (n=63) between levels of therapist-reported Mini-MACS and GMFCS. Mini-MACS, Mini-Manual Classification System; GMFCS, Gross Motor Function Classification System

**Figure 2:**
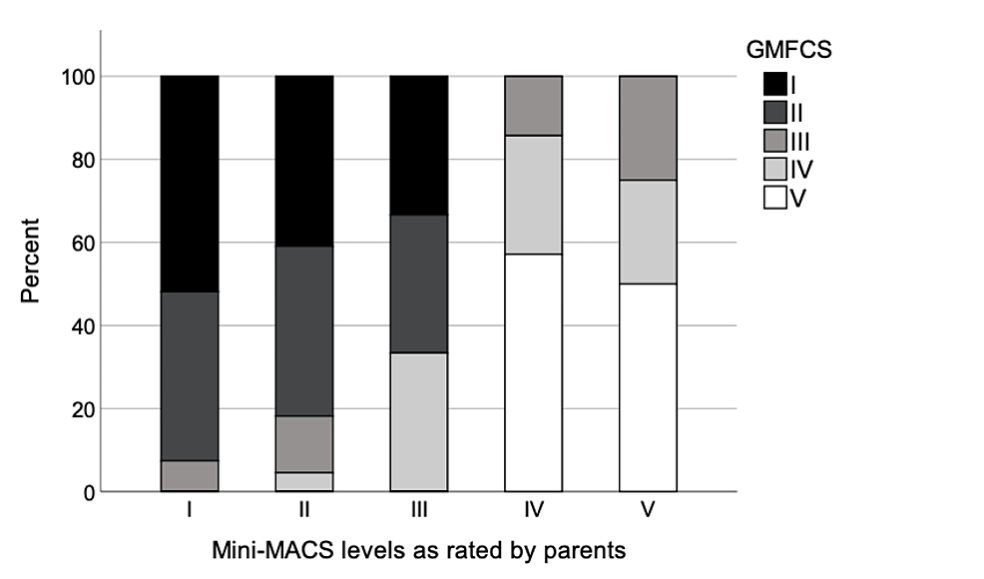
Percent distribution of the participated children (n=63) between levels of parent-reported Mini-MACS and GMFCS. Mini-MACS, Mini-Manual Classification System; GMFCS, Gross Motor Function Classification System

In addition, there was moderate to strong correlation between the Mini-MACS (initial ratings) and GMFCS with the parent- (ρ = 0.56, p < 0.0001) and therapist-reported (ρ = 0.64, p < 0.0001) Mini-MACS ratings (Table 3). Figures 1-2 show the percent distribution of the participants in the GMFCS levels in relation to therapist- and parent-reported Mini-MACS levels, respectively. 

**Table 3:** Distribution of Mini-MACS levels as rated by therapists and parents among GMFCS levels. Mini-MACS, Mini-Manual Ability Classification System; GMFCS, Gross Motor Function Classification System

1. Therapists	Mini-MACS ratings					
	I	II	III	IV	V	Total
GMFCS level						
I	14	9	1	–	–	24
II	9	11	1	–	–	21
III	1	4	–	–	2	7
IV	–	–	–	4	1	5
V	–	–	–	1	5	6
Total	24	24	2	5	8	63
2. Parents	Mini-MACS ratings					
	I	II	III	IV	V	Total
GMFCS level						
I	14	9	1	–	–	24
II	11	9	1	–	–	21
III	2	3	–	1	1	7
IV	–	1	1	2	1	5
V	–	–	–	4	2	6
Total	27	22	3	7	4	63

## Discussion

The Mini-MACS is a useful classification tool to describe how well young children aged between 1 and 4 years with CP use their hands to handle toys and age-relevant objects in activities of daily living. The Mini-MACS was developed in 2016 and has already been translated into 13 languages, including Greek, which indicates the clinicians’ realization and recognition of the importance of language to the classification procedure [19]. The findings of the present study indicated that the Greek version of the Mini-MACS was equivalent to the original English version in terms of meaning and context.

The Greek Mini-MACS demonstrated excellent test-retest reliability for both therapists and parents. This very high-reliability score found for the test-retest analysis (ICC = 0.97) established that the Greek Mini-MACS is a reliable scale when the same therapist or parent is applying it. Similar high levels of repeatability on both therapist and parent measures (ICC = 0.87-0.99) have been also shown in the Chinese version of Mini-MACS [20] as well as in the test-retest reliability studies of various linguistic versions of the MACS when used in children aged 4-18 years, including the Greek version [7, 21-23].

Additionally, the interrater reliability of the Greek Mini-MACS yielded acceptable agreement for ratings between therapists and parents involving children aged 12-50 months, which is virtually equal to that observed in the original Swedish (ICC = 0.90, 95% CI = 0.84-0.94) and Chinese versions (ICC = 0.92, 95% CI = 0.86-0.95) of Mini-MACS [9, 20]. In cases where children were classified to a different level by their parent and therapist, 13 out of 18 parents (72%) classified their children’s manual ability as being higher functional level than therapists perceived to be. This is in contrast to most studies [24-25], including the original Mini-MACS study by Eliasson et al., indicating that parents are more inclined to allocate their children to levels of lower functional ability than therapists [9]. 

Parents interact with their children in various environments and with different situations (and not in a standardized clinical environment as is commonly the case with therapists), which helps contribute to a clearer and fuller picture of the children's motor performance. In spite of this, parents may overestimate, relative to therapists, their child’s motor abilities and skills [26], particularly in early childhood even in case of severe cerebral injury [27]. This tendency to overestimate may be due to parents’ reluctance to acknowledge that their child has a problem. Further research on interrater reliability among professionals and with caregivers is advisable in order to consider and understand the potential differences underlying parents’ and health professionals’ classifications.

In this study, the association between Mini-MACS and GMFCS was also investigated. As was also demonstrated by similar studies [6-7, 28], the study results indicated that there was a moderate to strong correlation between the Mini-MACS and GMFCS with the parent- and therapist-reported Mini-MACS scores. This slightly high correlation result, which most probably derives from the fact that children belonged to different types of CP [29], confirms and underlines the different construct that these two classification systems are built on and provide, fine versus gross motor function [6].

One possible limitation involves the sampling procedure. A sample of young children with CP from public and private pediatric rehabilitation units of the Athens metropolitan area was used. Thus, the sample may be considered not to be representative of the entire population of children with CP in Greece, as socioeconomic status, lifestyle, and culture adopted by the local communities could potentially influence the way parents perceive their children’s functional abilities and disabilities. Nevertheless, since Athens metropolitan area covers more than 36% of the Greek population with multi-cultural and multi-socioeconomic backgrounds [30], the study sample is rendered quite representative of the general population.

## Conclusions

In summary, the Greek version of the Mini-MACS proved to be comprehensible and easy to use by therapists and parents. The Greek Mini-MACS demonstrated excellent test-retest reliability and good interrater reliability between therapists and parents. A moderate-to-strong agreement between the Mini-MACS and GMFCS was also observed. More investigation would be important to better estimate the interrater reliability between health professionals (therapists and physicians) and parents and to better understand the variations that are likely to exist between the professional- and parent-reported classifications.
